# Adverse events associated with targeted therapy and immunotherapy for ovarian cancer: a FAERS pharmacovigilance study

**DOI:** 10.3389/fphar.2026.1867038

**Published:** 2026-06-23

**Authors:** Kui Yao, Hongling Peng

**Affiliations:** 1 Department of Gynecology and Obstetrics, West China Second University Hospital, Sichuan University, Chengdu, China; 2 Laboratory of Birth Defects and Related Diseases of Women and Children (Sichuan University), Ministry of Education, Chengdu, China

**Keywords:** adverse events, bevacizumab, disproportionality analysis, ovarian cancer, PARP inhibitors

## Abstract

**Background:**

The addition of targeted therapy and immunotherapy has significantly improved survival in ovarian cancer patients. This analysis aimed to investigate the adverse events associated with targeted and immunotherapy drugs in patients with ovarian cancer.

**Methods:**

Adverse event reports related to bevacizumab, poly (ADP-ribose) polymerase (PARP) inhibitors, and immune checkpoint inhibitors (ICI: PD-1, PD-L1, CTLA-4 inhibitors) in ovarian cancer were sourced from the FDA Adverse Event Reporting System (FAERS) database (Q3 2014 to Q3 2025). The disproportionality analysis, including the reporting odds ratio (ROR), proportional reporting ratio (PRR), and Bayesian confidence propagation neural network (BCPNN), was employed to detect safety signals associated with different drugs.

**Results:**

A total of 209,920 adverse event reports were included in the analysis, involving 33,538 ovarian cancer patients. Among adverse events associated with the target drug (n = 183,935), 157,470 (85.61%) reports were related to PARP inhibitors, followed by bevacizumab [21,001 (11.42%)], PD-1 inhibitors [3,702 (2.01%)], PD-L1 inhibitors [1,317 (0.72%)], and CTLA-4 inhibitors [425 (0.23%)]. Adverse events are most frequently reported within 30 days of taking the medication. The most frequently reported safety signals at the preferred term level associated with PARP inhibitors included Energy increased, Vitamin D decreased, Nocturia, Intentional underdose, Hunger, and Brain neoplasm. For bevacizumab, the most frequently reported safety signals were Gastrointestinal perforation, Proteinuria, Intestinal perforation, and Embolism. For ICI, the most frequently reported safety signals comprised Product use in unapproved indication, Off label use, Death, and Malignant neoplasm progression. The most frequently reported safety signals linked to combination therapy using different drugs vary. For the combination of bevacizumab and PARP inhibitors, the most frequently reported safety signals included Interstitial lung disease, Myelodysplastic syndrome, Myelosuppression, Acute myeloid leukaemia, and Proteinuria.

**Conclusion:**

This study systematically analyzed the safety signals related to different drugs during the treatment of ovarian cancer, which may assist clinicians in identifying drug alert signals.

## Introduction

Ovarian cancer is a common malignant tumor that affects the health of women. Epithelial ovarian cancer accounts for over 95% of ovarian cancers, with high-grade serous carcinoma being the most common subtype (Matulonis et al., 2016). Non-epithelial ovarian cancers constitute 5% of ovarian cancers, primarily including germ cell tumors and sex cord-stromal tumors, as well as rare small cell carcinomas and ovarian sarcomas (Torre et al., 2018). In 2022, more than 320,000 new cases of ovarian cancer were diagnosed worldwide, resulting in over 200,000 deaths and imposing a significant global disease burden (Bray et al., 2024). The overall survival rate for ovarian cancer patients varies significantly depending on tumor stage. The 5-year overall survival rate for patients in stage I is 92.1%, while it is only 25% for those in stages III and IV (Torre et al., 2018; Reid et al., 2017). However, more than two-thirds of ovarian cancer patients are diagnosed at an advanced stage (Lheureux et al., 2019b). Although platinum-based chemotherapy initially yields high response rates, high recurrence rates and platinum resistance remain major challenges affecting patient survival (Arend et al., 2020; Caruso et al., 2025).

Angiogenesis plays a pivotal role in tumor growth and metastasis. Bevacizumab, which targets the anti-vascular endothelial growth factor (VEGF) signaling pathway, inhibits tumor angiogenesis and reshapes the tumor microenvironment, making it a treatment option for ovarian cancer (Marth et al., 2017). Since bevacizumab was approved by the FDA in 2014 for the treatment of platinum-resistant recurrent ovarian cancer, it has become a first-line treatment option for advanced ovarian cancer, widely used in both newly diagnosed and recurrent ovarian cancer (Monk et al., 2025). Immune checkpoint inhibitors (ICI) and poly (ADP-ribose) polymerase (PARP) inhibitors also offer treatment options for ovarian cancer, with PARP inhibitors demonstrating improved progression-free survival (PFS) in both first-line and second-line treatments (O'Malley et al., 2023). The combination strategy of bevacizumab with ICI and PARP inhibitors has been employed in the treatment of ovarian cancer, aiming to overcome drug resistance through multidimensional synergistic therapy that integrates immune modulation, DNA repair intervention, and multi-target pathway inhibition, to prolong survival (Zhang et al., 2025b). Clinical trials demonstrate that the triple combination therapy of bevacizumab, durvalumab (anti-PD-L1), and olaparib (PARP inhibitors) prolongs PFS in homologous recombination deficiency-positive ovarian cancer (Harter et al., 2025). The combination of atezolizumab (anti-PD-L1), bevacizumab, and chemotherapy improved overall survival in PD-L1-positive patients, but it also increased the incidence of immune-related adverse events (Kurtz et al., 2023). The safety profile of first-line maintenance therapy with niraparib (PARP inhibitors) plus bevacizumab was consistent with the known safety profiles of niraparib and bevacizumab as monotherapies (Hardesty et al., 2022). The use of different medications may lead to different adverse events (Rawal et al., 2023). However, the adverse events associated with the use of these drugs, either individually or in combination, in the treatment of ovarian cancer patients have not yet been systematically analyzed.

The FDA Adverse Event Reporting System (FAERS) is an adverse event reporting system administered by the FDA and serves as a public database of spontaneously reported adverse drug reactions. Compared to adverse event data from clinical trials, FAERS provides data on adverse events from the real-world population, long-term medication use, and complex polypharmacy scenarios. Previous pharmacovigilance studies on ovarian cancer based on the FAERS database have focused on adverse events associated with PARP inhibitors (Yang et al., 2025b; Yang et al., 2025a). Herein, this study intended to comprehensively analyze adverse events related to bevacizumab, PARP inhibitors, ICI, and their combination regimens in ovarian cancer patients, to assist clinicians in identifying drug alert signals.

## Methods

### Data source and patients

Data on drug-related adverse events in ovarian cancer patients were sourced from the FAERS database between the third quarter of 2014 and the third quarter of 2025. FAERS is an internationally recognized and publicly accessible adverse event reporting database that contains adverse events from the real-world population (https://fis.fda.gov/extensions/FPD-QDE-FAERS/FPD-QDE-FAERS.html). This database collects spontaneous adverse event reports from various sources, including healthcare professionals, patients, pharmaceutical manufacturers, and other individuals. This disproportionality analysis included 45 quarters of American Standard Code for Information Interchange (ASCII) data packages, covering 7 parts: patient demographic and administrative information, drug information, adverse event information, patient medical outcomes, reporting source information, drug initiation and discontinuation information, and drug indications. Ovarian cancer patients were identified based on the information provided in the indication file (e.g., ovarian cancer, ovarian epithelial cancer). Detailed information on the identification of ovarian cancer can be found in Supplementary Table 1. This research used fully anonymized and de-identified patient data derived from a publicly available database, ethical approval and informed consent were exempted.

### Procedures

Drug information for ovarian cancer treatment is obtained from the DRUG file in the FAERS database. Both drug names and active pharmaceutical ingredients are used to screen target drugs: Bevacizumab (Avastin), CTLA-4 inhibitors [ipilimumab (Yervoy), tremelimumab (Imjudo)], PD-1 inhibitors [pembrolizumab (Keytruda), nivolumab (Opdivo), Cemiplimab (Libtayo), Dostarlimab (Jemperli), Toripalimab (Loqtorzi), Tislelizumab (Tevimbra), Sintilimab, Camrelizumab, Penpulimab, Zimberelimab, Serplulimab], PD-L1 inhibitors [atezolizumab (Tecentriq), durvalumab (Imfinzi), Avelumab (Bavencio), Envafolimab, Sugemalimab (Cejemly)], LAG-3 inhibitors [Relatlimab], PD-1/CTLA-4 inhibitors [Cadonilimab], PD-1/LAG-3 inhibitors [Opdualag], and PARP inhibitors [Olaparib (Lynparza), niraparib (Zejula), rucaparib (Rubraca)]. The role codes for drugs in adverse events are categorized into four types: Primary Suspect Drug (PS), Secondary Suspected Drug (SS), Concomitant (C), and Interaction (I). PS is the main drug responsible for the adverse event. If the ROLE_COD of the target drug is PS and the other drugs are not SS/C/I, it is considered monotherapy. If the ROLE_COD of the target drug is PS and the other drugs are SS/C/I, it is considered a combination therapy. In this analysis, we utilized bevacizumab, ICI [CTLA-4, PD-1, PD-L1, LAG-3 inhibitors], and PARP inhibitors to identify cases within the DRUG file and designated the role code as PS to improve identification accuracy. Moreover, an imbalance ratio measurement method was applied to identify genuine “drug adverse event” signals with the aim of decreasing the false positive rate.

For adverse events, the preferred term (PT) from the Medical Dictionary for Regulatory Activities (MedDRA) was used to code adverse events in each report, and the corresponding primary system organ classes (SOC) for these PTs were provided. Within each SOC, the PT count was employed to describe the incidence of each adverse event corresponding to significant safety signals, allowing for the identification of the most prevalent adverse event for each drug in a given SOC. This study did not conduct a manual review of MedDRA preferred terms to exclude non-clinical reporting terms. Furthermore, the characteristics of the patients were collected, covering age, weight, occupation types of adverse event reporters, reporter country, stage, histological subtype, recurrence, route, and outcomes. For characteristic variables with missing values, the missing values were classified into the “unknown” group.

### Statistical analysis

This analysis strictly follows the data cleaning guidelines on the FDA website, removing duplicate reports or reports requiring deletion. Following the FDA’s recommended method for removing duplicate reports, select the “PRIMARYID”, “CASEID”, and “FDA_DT” fields from the DEMO table. Sort the reports with the same “CASEID” in the order of “CASEID”, “FDA_DT”, and “PRIMARYID”, retaining the report with the largest “FDA_DT” value. If both “CASEID” and “FDA_DT” are identical, retain the report with the largest “PRIMARYID” value. Additionally, starting from the first quarter of 2019, each quarterly data package includes a list of reports to be deleted. After removing duplicates, exclude these reports based on the “CASEID” listed in the deletion report list.

For this pharmacovigilance analysis, a disproportionality analysis was conducted to contrast the proportion of particular adverse reactions linked to one or more drugs with those reported for the same agents throughout the whole database. To enhance the robustness of signal detection for drug-associated adverse events, we adopted three complementary disproportionality analysis methods: the reporting odds ratio (ROR), proportional reporting ratio (PRR), and Bayesian confidence propagation neural network (BCPNN) (Zhang et al., 2025a). ROR is an assessment of the relationship between a specific adverse event and a specific drug, and it is compared with all other drugs in the database (Kerr et al., 2023). A signal is considered present if the lower 95% confidence interval (CI) of the ROR exceeds 1 and there are at least 3 reports of suspected drug-related adverse reactions. PRR determines the incidence rate of adverse events related to exposure to a specific drug by analyzing the proportion of adverse drug events among exposed individuals compared to unexposed individuals (Noguchi et al., 2020). A signal is considered present if the PRR ≥3, or if the 95%CI (lower bound) of the PRR ≥1 and the PRR ≥2. BCPNN determines the correlation between drugs and adverse reactions by calculating the information content (IC) (Tada et al., 2020). If the IC-2SD in BCPNN is > 0, a signal is considered to be present.

Furthermore, a multivariable logistic analysis was applied to explore the factors that affect hospitalization or mortality due to adverse events. The multivariable logistic analysis was adjusted for all covariates (age, weight, occupation types of adverse event reporters, reporter country, stage, histological subtype, recurrence, and drug type), whereas the exposure of interest was excluded from covariates in its corresponding analysis. Multicollinearity tests and calibration curves were used to evaluate the model. Statistical analyses were carried out with R software version 4.5.1 (Institute for Statistics and Mathematics, Vienna, Austria), with a P-value <0.05 regarded as statistically significant.

## Results

### Descriptive analysis of the adverse event reports

**FIGURE 1:**
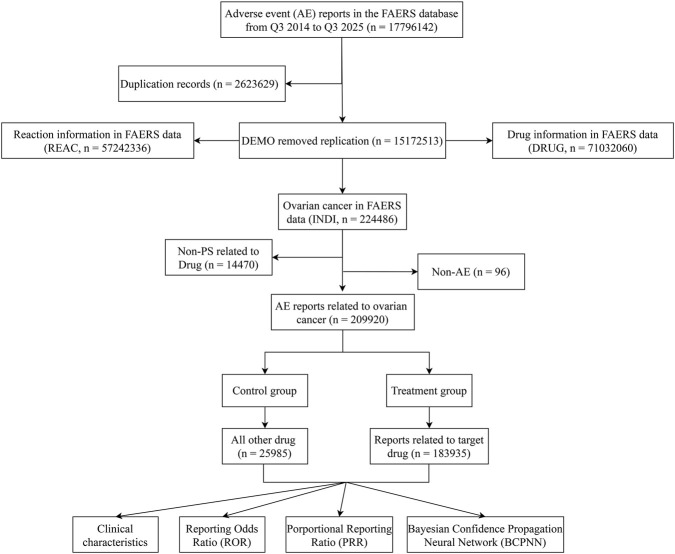
Flowchart of the study design.

**FIGURE 2:**
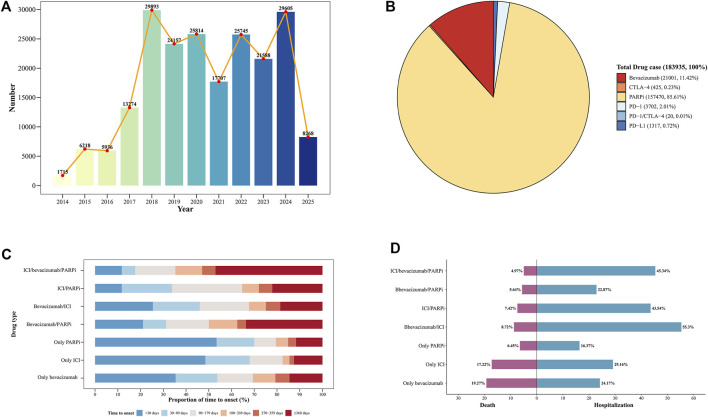
General characteristics of drugs-related adverse events in patients with ovarian cancer. **(A)** The trend of the incidence rate of adverse events; **(B)** The proportion of adverse events related to different drugs; **(C)** The distribution of the occurrence time of adverse events; **(D)** Deaths and hospitalizations caused by adverse events related to different drugs. ICI, immune checkpoint inhibitors; PARPi, poly (ADP-ribose) polymerase inhibitors; CTLA-4, cytotoxic T lymphocyte antigen; PD-1, programmed death 1; PD-L1, programmed death ligand-1.

A total of 224,486 adverse event reports associated with ovarian cancer were recorded in the FAERS database from 2014 to 2025. After screening, 209,920 reports were included in the analysis (Figure 1). The incidence of adverse events related to ovarian cancer rose steadily from 2015 to 2018, peaking at 29,893 cases in 2018, followed by 29,605 cases in 2024 (Figure 2A). Among adverse events associated with the target drug (n = 183,935), PARP inhibitors accounted for 157,470 (85.61%) cases, followed by bevacizumab [21,001 (11.42%)], PD-1 inhibitors [3,702 (2.01%)], PD-L1 inhibitors [1,317 (0.72%)], and CTLA-4 inhibitors [425 (0.23%)] (Figure 2B). For the occurrence time of adverse events, over 50% PARP inhibitors-related adverse events, over 30% bevacizumab-related adverse events, and nearly 50% ICI-related adverse events may occur within 30 days (Figure 2C). In terms of hospitalization and mortality rates, PARP inhibitor-related adverse events resulted in hospitalization for 16.37% of patients and death for 6.45% of patients (Figure 2D). Bevacizumab-related adverse events led to hospitalization for 24.17% of patients and death for 19.27% of patients. ICI-related adverse events caused hospitalization for 29.16% of patients and death for 17.22% of patients.

Table 1 presents the characteristics of 33,538 ovarian cancer patients from 209,920 adverse event reports. The age distribution revealed that adverse events occurred most commonly in the 40–64-year group [7,382 (22.01%)], followed by the >64-year [6,391 (19.06%)] and 18–39-year [335 (1.00%)] groups, while 19,430 (57.93%) patients did not show age information. In terms of reporter occupations for adverse events, 14,823 (44.20%) reports were submitted by consumers, followed by physicians [9,568 (28.53%)], health professionals [3,295 (9.82%)], and other health-professionals [3,209 (9.57%)]. Adverse event reports originated from 103 countries, with the United States [22,514 (67.13%)] accounting for the highest proportion of reported adverse events, followed by Japan [3,606 (10.75%)], Canada [928 (2.77%)], France [826 (2.46%)], and China [832 (2.48)]. There were 12,075 (21.38%) reports involving hospitalizations, 4,580 (8.11%) reports involving deaths, and 24,275 (42.98%) reports involving other serious events.

**TABLE 1:** The characteristics of ovarian cancer patients.

Variables	Total	Bevacizumab only	ICI only	PARPi only	Bevacizumab and ICI	ICI and PARPi	Bevacizumab and PARPi	ICI, bevacizumab, and PARPi
Age, n (%)								
18–39 years	335 (1.00)	120 (2.85)	28 (3.37)	144 (0.54)	17 (3.20)	10 (3.40)	13 (1.20)	3 (2.68)
40–64 years	7382 (22.01)	1512 (35.97)	301 (36.22)	4855 (18.33)	234 (43.98)	102 (34.69)	331 (30.54)	47 (41.96)
>64 years	6391 (19.06)	1049 (24.95)	215 (25.87)	4482 (16.93)	204 (38.35)	97 (32.99)	310 (28.60)	34 (30.36)
Unknown	19430 (57.93)	1523 (36.23)	287 (34.54)	17000 (64.20)	77 (14.47)	85 (28.91)	430 (39.67)	28 (25.00)
Weight, n (%)								
<50 kg	731 (2.18)	209 (4.97)	43 (5.17)	394 (1.49)	30 (5.64)	13 (4.42)	40 (3.69)	2 (1.79)
50–100 kg	4649 (13.86)	1148 (27.31)	219 (26.35)	2667 (10.07)	246 (46.24)	122 (41.50)	203 (18.73)	44 (39.29)
>100 kg	324 (0.97)	56 (1.33)	18 (2.17)	224 (0.85)	8 (1.50)	7 (2.38)	7 (0.65)	4 (3.57)
Unknown	27834 (82.99)	2791 (66.39)	551 (66.31)	23196 (87.59)	248 (46.62)	152 (51.70)	834 (76.94)	62 (55.36)
Occupation types of AE reporters, n (%)								
Consumer	14823 (44.20)	670 (15.94)	240 (28.88)	13542 (51.14)	12 (2.26)	79 (26.87)	276 (25.46)	4 (3.57)
Health professional	3295 (9.82)	674 (16.03)	167 (20.10)	2166 (8.18)	90 (16.92)	74 (25.17)	102 (9.41)	22 (19.64)
Physician	9568 (28.53)	2270 (54.00)	285 (34.30)	5821 (21.98)	413 (77.63)	109 (37.07)	589 (54.34)	81 (72.32)
Other health-professional	3209 (9.57)	272 (6.47)	100 (12.03)	2782 (10.51)	10 (1.88)	22 (7.48)	19 (1.75)	4 (3.57)
Pharmacist	858 (2.56)	302 (7.18)	20 (2.41)	486 (1.84)	1 (0.19)	3 (1.02)	46 (4.24)	0 (0.00)
Lawyer	4 (0.01)	1 (0.02)	2 (0.24)	1 (0.00)	0 (0.00)	0 (0.00)	0 (0.00)	0 (0.00)
Unknown	1781 (5.31)	15 (0.36)	17 (2.05)	1683 (6.36)	6 (1.13)	7 (2.38)	52 (4.80)	1 (0.89)
Reporter country, n (%)								
United States	22514 (67.13)	1411 (33.56)	557 (67.03)	19742 (74.55)	177 (33.27)	180 (61.22)	406 (37.45)	41 (36.61)
Japan	3606 (10.75)	525 (12.49)	55 (6.62)	2712 (10.24)	20 (3.76)	10 (3.40)	277 (25.55)	7 (6.25)
Canada	928 (2.77)	126 (3.00)	14 (1.68)	770 (2.91)	2 (0.38)	3 (1.02)	9 (0.83)	4 (3.57)
France	826 (2.46)	188 (4.47)	23 (2.77)	353 (1.33)	109 (20.49)	9 (3.06)	115 (10.61)	29 (25.89)
China	832 (2.48)	283 (6.73)	34 (4.09)	422 (1.59)	45 (8.46)	1 (0.34)	44 (4.06)	3 (2.68)
Others	4832 (14.41)	1671 (39.75)	148 (17.81)	2482 (9.37)	179 (33.65)	91 (30.95)	233 (21.49)	28 (25.00)
Stage, n (%)								
Stage I	10 (0.03)	7 (0.17)	0 (0.00)	3 (0.01)	0 (0.00)	0 (0.00)	0 (0.00)	0 (0.00)
Stage II	20 (0.06)	4 (0.10)	0 (0.00)	14 (0.05)	0 (0.00)	0 (0.00)	0 (0.00)	2 (1.79)
Stage III	331 (0.99)	81 (1.93)	11 (1.32)	211 (0.80)	7 (1.32)	2 (0.68)	19 (1.75)	0 (0.00)
Stage IV	644 (1.92)	201 (4.78)	73 (8.78)	315 (1.19)	9 (1.69)	10 (3.40)	32 (2.95)	4 (3.57)
Ovarian cancer, NOS	32533 (97.00)	3911 (93.03)	747 (89.89)	25938 (97.95)	516 (96.99)	282 (95.92)	1033 (95.30)	106 (94.64)
Histological subtype, n (%)								
*Ovarian germ cell cancer*	23 (0.07)	13 (0.31)	3 (0.36)	4 (0.02)	1 (0.19)	2 (0.68)	0 (0.00)	0 (0.00)
Ovarian germ cell cancer	23 (0.07)	13 (0.31)	3 (0.36)	4 (0.02)	1 (0.19)	2 (0.68)	0 (0.00)	0 (0.00)
*Ovarian epithelial cancer*	1358 (4.05)	398 (9.47)	73 (8.78)	761 (2.87)	58 (10.90)	22 (7.48)	40 (3.69)	6 (5.36)
Ovarian epithelial cancer	1241 (3.70)	356 (8.47)	42 (5.05)	742 (2.80)	37 (6.95)	22 (7.48)	37 (3.41)	5 (4.46)
Ovarian endometrioid	9 (0.03)	1 (0.02)	3 (0.36)	5 (0.02)	0 (0.00)	0 (0.00)	0 (0.00)	0 (0.00)
Borderline ovarian Tumour	3 (0.01)	3 (0.07)	0 (0.00)	0 (0.00)	0 (0.00)	0 (0.00)	0 (0.00)	0 (0.00)
Mucinous cystadenocarcinoma ovary	35 (0.10)	19 (0.45)	2 (0.24)	11 (0.04)	0 (0.00)	0 (0.00)	3 (0.28)	0 (0.00)
Ovarian clear cell carcinoma	70 (0.21)	19 (0.45)	26 (3.13)	3 (0.01)	21 (3.95)	0 (0.00)	0 (0.00)	1 (0.89)
*Ovarian granulosa cell tumour*	7 (0.02)	1 (0.02)	4 (0.48)	0 (0.00)	1 (0.19)	0 (0.00)	1 (0.09)	0 (0.00)
*Ovarian cancer, NOS*	32150 (95.86)	3792 (90.20)	751 (90.37)	25716 (97.11)	472 (88.72)	270 (91.84)	1043 (96.22)	106 (94.64)
Recurrence, n (%)								
No	31949 (95.26)	3975 (94.55)	801 (96.39)	25284 (95.48)	451 (84.77)	276 (93.88)	1051 (96.96)	111 (99.11)
Yes	1589 (4.74)	229 (5.45)	30 (3.61)	1197 (4.52)	81 (15.23)	18 (6.12)	33 (3.04)	1 (0.89)
Route, n (%)								
Oral	26982 (47.77)	661 (9.05)	221 (11.56)	24450 (58.28)	207 (14.67)	373 (36.64)	971 (38.82)	99 (25.78)
Intravenous (NOS)	4232 (7.49)	2118 (29.01)	696 (36.40)	230 (0.55)	517 (36.64)	267 (26.23)	281 (11.24)	123 (32.03)
Intravenous drip	1199 (2.12)	792 (10.85)	133 (6.96)	11 (0.03)	144 (10.21)	12 (1.18)	99 (3.96)	8 (2.08)
Others	1472 (2.61)	634 (8.68)	76 (3.97)	559 (1.33)	89 (6.31)	47 (4.62)	45 (1.80)	22 (5.73)
Unknown	22596 (40.01)	3097 (42.41)	786 (41.11)	16703 (39.81)	454 (32.18)	319 (31.34)	1105 (44.18)	132 (34.38)
Outcome, n (%)								
Other serious events	24275 (42.98)	2926 (40.07)	667 (34.88)	18645 (44.44)	315 (22.32)	324 (31.83)	1262 (50.46)	136 (35.42)
Hospitalization	12075 (21.38)	1979 (27.10)	658 (34.41)	7290 (17.38)	862 (61.09)	486 (47.74)	617 (24.67)	183 (47.66)
Death	4580 (8.11)	1416 (19.39)	317 (16.58)	2505 (5.97)	113 (8.01)	69 (6.78)	146 (5.84)	14 (3.65)
Life-threatening	2083 (3.69)	343 (4.70)	50 (2.62)	1339 (3.19)	78 (5.53)	57 (5.60)	174 (6.96)	42 (10.94)
Disability	347 (0.61)	110 (1.51)	11 (0.58)	157 (0.37)	20 (1.42)	15 (1.47)	31 (1.24)	3 (0.78)
Required intervention	14 (0.02)	2 (0.03)	0 (0.00)	12 (0.03)	0 (0.00)	0 (0.00)	0 (0.00)	0 (0.00)
Congenital anomaly	17 (0.03)	4 (0.05)	0 (0.00)	11 (0.03)	0 (0.00)	0 (0.00)	2 (0.08)	0 (0.00)
Unknown	13090 (23.18)	522 (7.15)	209 (10.93)	11994 (28.59)	23 (1.63)	67 (6.58)	269 (10.76)	6 (1.56)

ICI, immune checkpoint inhibitors; PARPi, poly (ADP-ribose) polymerase inhibitors; NOS, not otherwise specified; AE, adverse events.

### Disproportionality analysis of adverse events at the PT level

**FIGURE 3:**
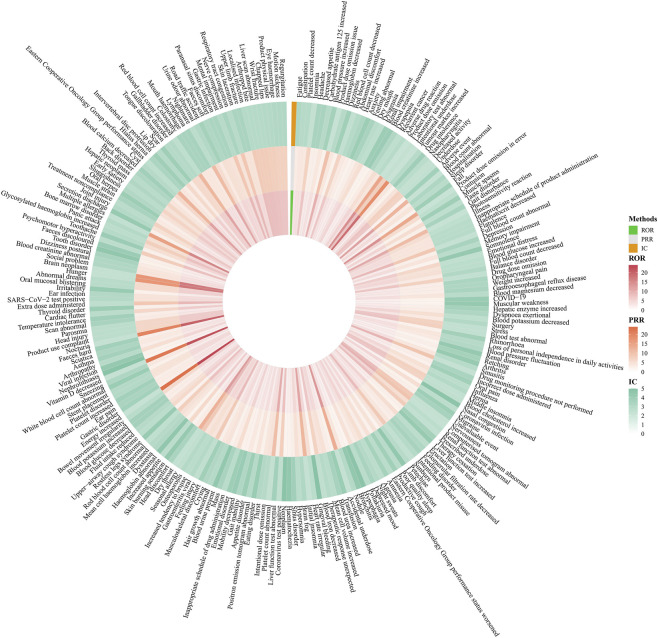
The circular graph of the signal for adverse events related to PARP inhibitors at the PT level. The darker the color of the three adverse event signal indicators (ROR, PRR, IC), the larger the value of the indicators. PARPi, poly (ADP-ribose) polymerase inhibitors; ROR, reporting odds ratio; PRR, proportional reporting ratio; IC, information content; PT, the preferred term.

The circular graph of the signal for 244 adverse events related to PARP inhibitors at the PT level is shown in Figure 3. The top 20 most frequently reported safety signals related to PARP inhibitors based on ROR values were (Supplementary Table 2): Energy increased, Vitamin D decreased, Nocturia, Intentional underdose, Hunger, Brain neoplasm, Multiple allergies, Product dose omission, Product dose omission in error, Accidental underdose, Dry throat, Eastern Cooperative Oncology Group performance status, Blood count abnormal, Initial insomnia, Red blood cell count increased, Nightmare, Photosensitivity reaction, Platelet count abnormal, Product dose omission issue, and Computerised tomogram abnormal.

**FIGURE 4:**
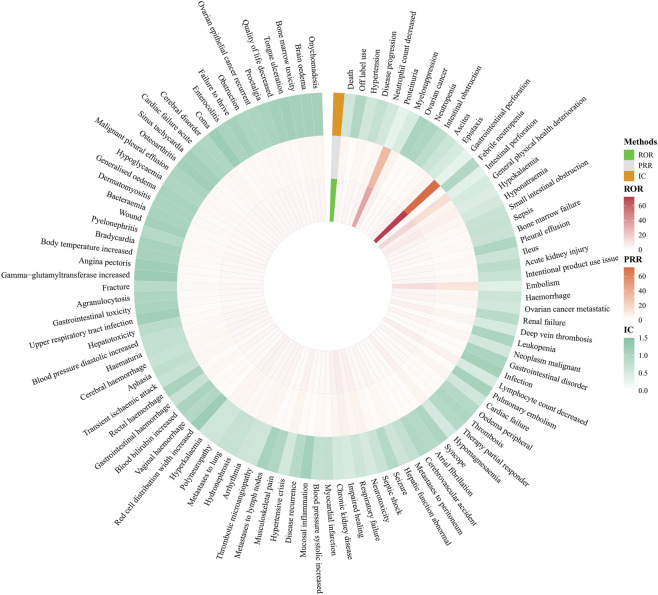
The circular graph of the signal for adverse events correlated with BEVACIZUMAB at the PT level. The darker the color of the three adverse event signal indicators (ROR, PRR, IC), the larger the value of the indicators. ROR, reporting odds ratio; PRR, proportional reporting ratio; IC, information content; PT, the preferred term.

Figure 4 lists the circular graph of the signal for 106 adverse events correlated with bevacizumab at the PT level. The most frequently reported safety signals based on ROR values include (Supplementary Table 3): Gastrointestinal perforation, Proteinuria, Intestinal perforation, Embolism, Hypokalaemia, Impaired healing, Lymphocyte count decreased, Neurotoxicity, Myelosuppression, Deep vein thrombosis, Hypertensive crisis, Hypomagnesaemia, General physical health deterioration, Chronic kidney disease, Epistaxis, Polyneuropathy, Hyponatraemia, Hyperkalaemia, Neoplasm malignant, and Thrombotic microangiopathy.

**FIGURE 5:**
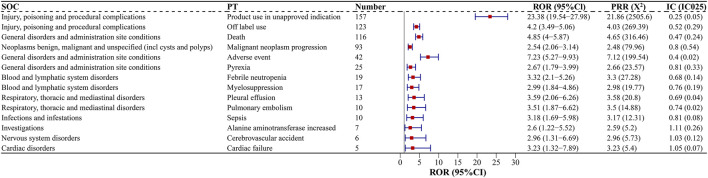
The forest plot of adverse events related to ICI monotherapy. ICI, immune checkpoint inhibitors; ROR, reporting odds ratio; PRR, proportional reporting ratio; IC, information content; PT, the preferred term; SOC, system organ classes.

The forest plot of 14 adverse events related to ICI monotherapy is demonstrated in Figure 5. These safety signals based on ROR values at the PT level were: Product use in unapproved indication, Off label use, Death, Malignant neoplasm progression, Adverse event, Pyrexia, Febrile neutropenia, Myelosuppression, Pleural effusion, Pulmonary embolism, Sepsis, Alanine aminotransferase increased, Cerebrovascular accident, and Cardiac failure.

**FIGURE 6:**
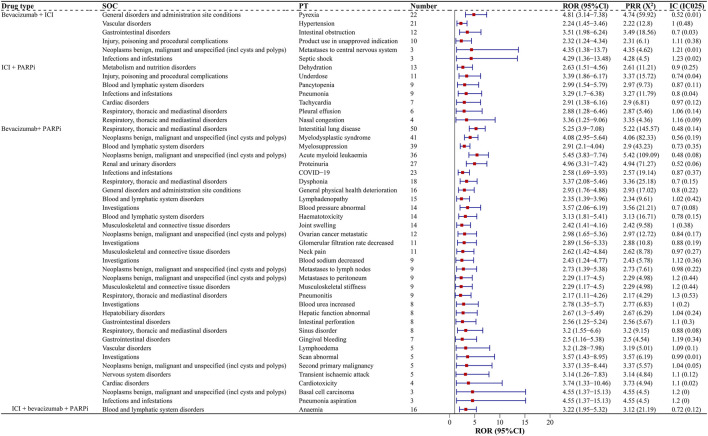
The forest plot of adverse events associated with different drug combination treatments. ICI, immune checkpoint inhibitors; PARPi, poly (ADP-ribose) polymerase inhibitors; ROR, reporting odds ratio; PRR, proportional reporting ratio; IC, information content; PT, the preferred term; SOC, system organ classes.

Figure 6 presents a forest plot of adverse events associated with different drug combination treatments. The most frequently reported safety signals based on ROR values related to the combination therapy of bevacizumab and ICI were: Pyrexia, Hypertension, Intestinal obstruction, Product use in unapproved indication, Metastases to central nervous system, and Septic shock. The most frequently reported safety signals correlated with combination therapy of PARP inhibitors and ICI include: Dehydration, Underdose, Pancytopenia, Pneumonia, Tachycardia, Pleural effusion, and Nasal congestion. The most frequently reported safety signals related to combination therapy of bevacizumab and PARP inhibitors were: Interstitial lung disease, Myelodysplastic syndrome, Myelosuppression, Acute myeloid leukaemia, Proteinuria, COVID-19, Dysphonia, General physical health deterioration, Lymphadenopathy, Blood pressure abnormal, Haematotoxicity, Joint swelling, Ovarian cancer metastatic, Glomerular filtration rate decreased, and Neck pain. The most frequently reported safety signals related to the combination therapy of PARP inhibitors, bevacizumab, and ICI were Anaemia.

**FIGURE 7:**
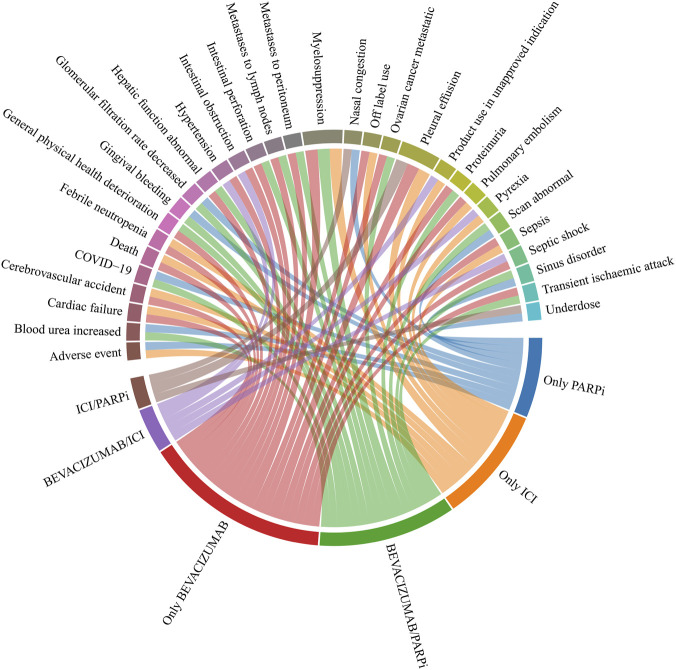
The correspondence between drug combinations and adverse events at the PT level. The lower half of the figure represents individual and combined treatments, while the upper half represents associated PTs. The connecting lines between the upper and lower halves indicate significant signals generated by treatment-associated PTs. Different treatment regimens and associated PTs are distinguished by color, and each associated PT has connecting lines extending from at least two treatment regimens. ICI, immune checkpoint inhibitors; PARPi, poly (ADP-ribose) polymerase inhibitors; PT, the preferred term.

Figure 7 illustrates the correspondence between drug combinations and adverse events (at the PT level). The three treatment options associated with the adverse event Myelosuppression were bevacizumab only, ICI only, and bevacizumab combined with PARP inhibitors, while the three treatment options related to the adverse event Pleural effusion were bevacizumab only, ICI only, and ICI combined with PARP inhibitors.

**FIGURE 8:**
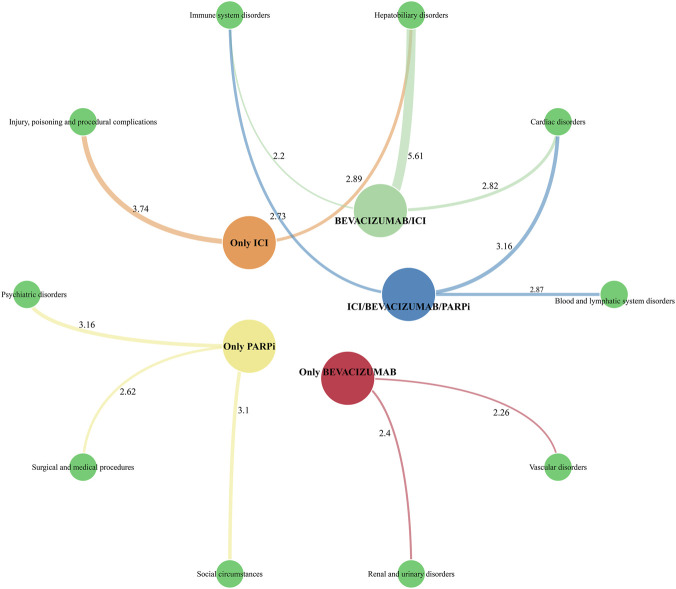
The 10 most relevant the system organ classification (SOC) corresponding to the 5 treatment modalities. The internal nodes represent 5 treatment modalities, while the external nodes represent 10 relevant SOCs. The lines connecting the internal nodes to the external nodes indicate the positive signals generated by the SOCs associated with those internal nodes. Each line is labeled with the actual ROR value, and the thickness of the line also reflects the magnitude of that ROR value. ICI, immune checkpoint inhibitors; PARPi, poly (ADP-ribose) polymerase inhibitors; ROR, reporting odds ratio.

Figure 8 shows the 10 most relevant SOCs corresponding to the 5 treatment modalities. The most frequently implicated SOCs for adverse events associated with different drugs were as follows: PARP inhibitors (psychiatric disorders, social circumstances, surgical and medical procedures), bevacizumab (renal and urinary disorders, vascular disorders), ICI (injury, poisoning and procedural complications, hepatobiliary disorders), the combination of bevacizumab and ICI (hepatobiliary disorders, cardiac disorders, immune system disorders), and the combination of bevacizumab, ICI, and PARP inhibitors (cardiac disorders, immune system disorders, blood and lymphatic system disorders).

The adverse events associated with different drug uses are presented across different stages (Supplementary Figures 1, 2), histological subtypes of patients (Supplementary Figure 3), and patients with recurrent (Supplementary Figure 4).

**FIGURE 9:**
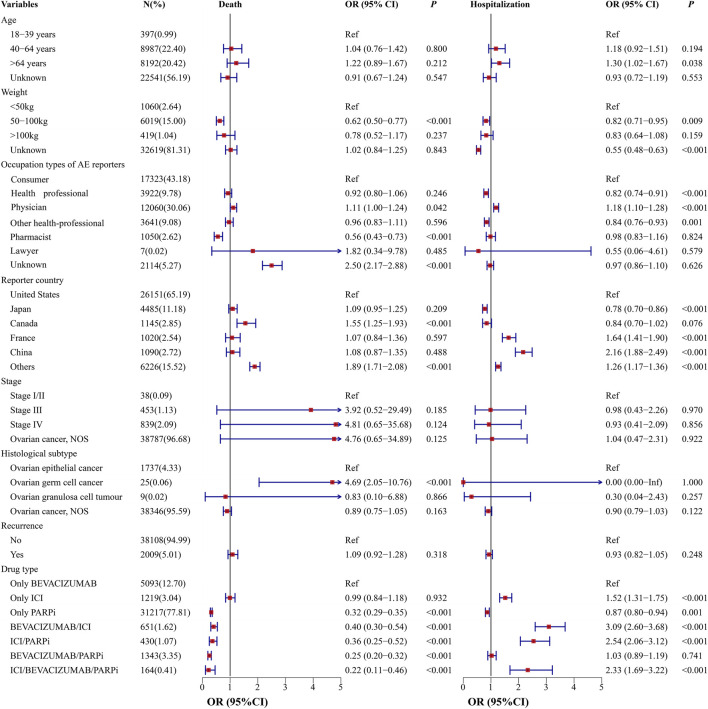
The multivariate logistic regression analysis for the factors influencing hospitalization or death due to adverse events. ICI, immune checkpoint inhibitors; PARPi, poly (ADP-ribose) polymerase inhibitors.

Additionally, Figure 9 presents the results of a multivariate logistic regression analysis examining the factors influencing hospitalization or death due to adverse events. Patients weighing 50–100 kg may have a lower death risk compared to those weighing <50 kg. Adverse event reports from pharmacists may be related to a lower death risk than those from consumers. Compared with reports from the United States, reports from Canada may be linked to a higher risk of death. Ovarian germ cell cancer may be correlated with a higher death risk than ovarian epithelial cancer. Furthermore, PARP inhibitors alone, the combination of bevacizumab and ICI, the combination of ICI and PARP inhibitors, the combination of bevacizumab and PARP inhibitors, and the combination of ICI, bevacizumab, and PARP inhibitors may be linked to a lower death risk compared to bevacizumab alone. The multicollinearity test indicated that there was no multicollinearity among the variables included in the multivariate logistic regression model (Supplementary Table 4). The calibration curve and the deviance residual plot demonstrated that the multivariate logistic regression model fits well (Supplementary Figure 5).

## Discussion

This study analyzed the adverse events associated with drug therapy in ovarian cancer patients. The most frequently reported safety signals at the PT level related to PARP inhibitors included Energy increased, Vitamin D decreased, Nocturia, Intentional underdose, Hunger, and Brain neoplasm. Those linked to bevacizumab comprised Gastrointestinal perforation, Proteinuria, Intestinal perforation, and Embolism. For ICI, the relevant safety signals were Product use in unapproved indication, Off label use, Death, and Malignant neoplasm progression. The safety signals associated with combination therapy using different drugs vary. The common safety signals linked to bevacizumab combined with PARP inhibitors include Interstitial lung disease, Myelodysplastic syndrome, Myelosuppression, Acute myeloid leukaemia, and Proteinuria.

Early-stage ovarian cancer is curable in 90% of patients, even in cases of the more aggressive invasive epithelial ovarian cancer (Peres et al., 2019; Lheureux et al., 2019a). However, approximately 75% of patients are already in stage III or IV at the time of diagnosis (Lheureux et al., 2019a). The conventional treatment for ovarian cancer patients includes surgery, chemotherapy, and radiation therapy. The addition of targeted therapy (e.g., bevacizumab, PARP inhibitors) and immunotherapy (e.g., ICI) has significantly improved patient survival (Lheureux et al., 2019a; Konstantinopoulos and Matulonis, 2023). The current study investigated the adverse events that occurred in ovarian cancer patients after they received targeted therapy and immunotherapy drugs. Among these adverse events, those related to PARP inhibitors accounted for 85.61%, followed by bevacizumab (11.42%), PD-1 inhibitors (2.01%), and PD-L1 inhibitors (0.72%). PARP enzymes (e.g., PARP-1, PARP-2) play a crucial role in repairing DNA single-strand breaks (Helleday et al., 2008). Inhibition of PARP leads to the accumulation of single-strand breaks, which in turn causes replication fork stalling and the accumulation of double-strand breaks. By trapping PARP, PARP inhibitors anchor PARP1 or PARP2 to damaged DNA loci and preclude the recruitment of additional DNA repair proteins. The accumulation of unrepaired DNA breaks results in cell death ultimately (LaFargue et al., 2019). Ovarian cancer patients harboring BRCA1/BRCA2 mutations or other homologous recombination deficiencies exhibit particular sensitivity to PARP inhibitors (Farmer et al., 2005). Clinical trials indicate that PARP inhibitors (Olaparib) in the maintenance treatment of advanced ovarian cancer patients achieved a 60% female PFS rate at 3 years, while in the placebo group it was only 27% (Moore et al., 2018). Our results demonstrated that the most frequently reported safety signals associated with PARP inhibitors included Energy increased, Vitamin D decreased, Nocturia, Intentional underdose, Hunger, Brain neoplasm, and Multiple allergies. The toxicity characteristics of PARP inhibitors are mainly reported as bone marrow suppression and anemia (Ngoi et al., 2021). Abnormal blood counts and platelet counts are also among the common adverse events related to PARP inhibitors in our analysis. Previous studies have reported major adverse cardiovascular events (Yang et al., 2025a) and peripheral neuropathy (Yang et al., 2025b) associated with the use of PARP inhibitors. Our findings suggest that multiple allergies may also be an adverse event that needs to be monitored when using PARP inhibitors. Moreover, over 50% PARP inhibitor-related adverse events may occur within 30 days. Adverse events occurring within the first month of PARP inhibitor use typically include gastrointestinal adverse reactions (e.g., nausea, vomiting, diarrhea, and loss of appetite) and neurological adverse events (e.g., fatigue, headache, dizziness, and insomnia), while hematologic toxicities (e.g., thrombocytopenia and anemia) usually occur 1 month or later (O'Malley et al., 2023; LaFargue et al., 2019).

Bevacizumab is a humanized monoclonal antibody targeting VEGF. The excessive VEGF in ovarian cancer, such as the adverse prognosis associated with angiogenesis/microvascular density and the ascites caused by excessive VEGF-induced capillary leakage, can benefit from treatment with bevacizumab (Lheureux et al., 2019a). Clinical trials have demonstrated that patients who use and maintain bevacizumab simultaneously have significantly improved PFS (Burger et al., 2011; Perren et al., 2011). Moreover, for patients who relapse after receiving bevacizumab as the first-line treatment, bevacizumab treatment can further improve PFS (Pignata et al., 2021). Our findings revealed that common safety signals linked to bevacizumab comprised Gastrointestinal perforation, Proteinuria, Intestinal perforation, and Embolism. Previous studies have reported additional toxicities associated with bevacizumab, including delayed wound healing, hypertension, and intestinal perforation (Lheureux et al., 2019a). For the occurrence time of adverse events, more than 30% bevacizumab-related adverse events occurred within 30 days. Additionally, common adverse events related to the combined use of bevacizumab and PARP inhibitors include Interstitial lung disease, Myelodysplastic syndrome, Myelosuppression, Acute myeloid leukaemia, and Proteinuria. Compared with placebo plus bevacizumab, PARP inhibitors (Olaparib) plus bevacizumab improved 5-year overall survival in patients with advanced ovarian cancer (Ray-Coquard et al., 2019; Ray-Coquard et al., 2023).

Immunotherapy with ICIs has transformed the treatment of multiple solid tumor types. However, the improvement in survival rates for ovarian cancer patients through ICIs treatment is very limited (Ghisoni et al., 2024). Although both PD-1 and PD-L1 are expressed in ovarian cancer, the response rate to monotherapy with ICI targeting PD-1/PD-L1 or CTLA-4 is approximately 10%–15% (Ghisoni et al., 2024). Our results demonstrated that adverse events associated with PD-1 inhibitors accounted for 2.01%, 0.72% for PD-L1 inhibitors, and 0.23% for CTLA-4 inhibitors. The common safety signals correlated with ICI included Product use in unapproved indication, Off label use, Death, and Malignant neoplasm progression. Additionally, the combination of ICI with bevacizumab and PARP inhibitors demonstrated some efficacy but did not significantly improve PFS in ovarian cancer patients (Ghisoni et al., 2024; Liu et al., 2019; Konstantinopoulos et al., 2019; Färkkilä et al., 2020). The common adverse events related to the combination of ICI and other drugs also differ. Common adverse events for the combination of ICI and bevacizumab include Pyrexia, Hypertension, and Intestinal obstruction, while common adverse events for the combination of ICI and PARP inhibitors include Dehydration, Underdose, Pancytopenia, and Pneumonia.

This study, based on the big data from FAERS, explored the drug-related adverse events of bevacizumab, PARP inhibitors, ICI, and their combination therapies in the treatment of ovarian cancer patients, providing evidence for the safety of different targeted and immunotherapeutic drugs in ovarian cancer. Nevertheless, several limitations should be considered. First, the data in the FAERS database are derived from spontaneous reports submitted by various countries and different types of reporters. The incompleteness of the data reporting may lead to bias. Second, due to the absence of baseline characteristics or previous risk factors as confounding factors, it is impossible to draw causal inferences regarding the correlation between the drug and adverse events. Third, the limited number of adverse event reports for the triple combination therapy or certain subgroups has impacted the statistical power for signal detection. Fourth, the definition of combination therapy used in our study (if a target drug is PS and other drugs are SS/C/I, it is considered combination therapy) may overestimate the actual incidence of concurrent use, as some secondary suspected drugs may not be taken simultaneously. Due to the severe lack of medication usage time data in the database, we also cannot estimate the combined medication situation based on the medication time. Fifth, more than 95% of patients had unknown ovarian cancer staging and histological subtypes. We cannot determine whether the observed significant between-drug differences in hospitalization and mortality rates are attributable to variations in patients’ baseline disease status.

## Conclusion

This disproportionality analysis investigated the adverse events related to targeted and immunotherapy drugs in ovarian cancer patients. Adverse events associated with PARP inhibitors were reported most frequently, followed by those related to bevacizumab. Most of these adverse events are likely to occur within 30 days after drug administration. This study may assist clinicians in identifying drug alert signals and may provide a reference for medication selection and safety monitoring in ovarian cancer patients. Furthermore, these findings are hypothesis-generating and require prospective validation.

## Data Availability

The original contributions presented in the study are included in the article/Supplementary Material, further inquiries can be directed to the corresponding author.

## References

[B1] Arend R. Westin S. N. Coleman R. L. (2020). Decision analysis for secondline maintenance treatment of platinum sensitive recurrent ovarian cancer: a review. Int. J. Gynecol. Cancer 30 (5), 684–694. 10.1136/ijgc-2019-001041 32079709

[B2] Bray F. Laversanne M. Sung H. Ferlay J. Siegel R. L. Soerjomataram I. (2024). Global cancer statistics 2022: GLOBOCAN estimates of incidence and mortality worldwide for 36 cancers in 185 countries. CA Cancer J. Clin. 74 (3), 229–263. 10.3322/caac.21834 38572751

[B3] Burger R. A. Brady M. F. Bookman M. A. Fleming G. F. Monk B. J. Huang H. (2011). Incorporation of bevacizumab in the primary treatment of ovarian cancer. N. Engl. J. Med. 365 (26), 2473–2483. 10.1056/NEJMoa1104390 22204724

[B4] Caruso G. Weroha S. J. Cliby W. (2025). Ovarian cancer: a review. Jama 334 (14), 1278–1291. 10.1001/jama.2025.9495 40690248

[B5] FäRKKILä A. Gulhan D. C. Casado J. Jacobson C. A. Nguyen H. Kochupurakkal B. (2020). Immunogenomic profiling determines responses to combined PARP and PD-1 inhibition in ovarian cancer. Nat. Commun. 11 (1), 1459. 10.1038/s41467-020-15315-8 32193378 PMC7081234

[B6] Farmer H. Mccabe N. Lord C. J. Tutt A. N. Johnson D. A. Richardson T. B. (2005). Targeting the DNA repair defect in BRCA mutant cells as a therapeutic strategy. Nature 434 (7035), 917–921. 10.1038/nature03445 15829967

[B7] Ghisoni E. Morotti M. Sarivalasis A. Grimm A. J. Kandalaft L. Laniti D. D. (2024). Immunotherapy for ovarian cancer: towards a tailored immunophenotype-based approach. Nat. Rev. Clin. Oncol. 21 (11), 801–817. 10.1038/s41571-024-00937-4 39232212

[B8] Hardesty M. M. Krivak T. C. Wright G. S. Hamilton E. Fleming E. L. Belotte J. (2022). OVARIO phase II trial of combination niraparib plus bevacizumab maintenance therapy in advanced ovarian cancer following first-line platinum-based chemotherapy with bevacizumab. Gynecol. Oncol. 166 (2), 219–229. 10.1016/j.ygyno.2022.05.020 35690498

[B9] Harter P. Trillsch F. Okamoto A. Reuss A. Kim J. W. Rubio-PéREZ M. J. (2025). Durvalumab with carboplatin/paclitaxel and bevacizumab followed by durvalumab and bevacizumab with or without olaparib maintenance in newly diagnosed non-BRCA-mutated advanced ovarian cancer. Ann. Oncol. 37, 503–520. 10.1016/j.annonc.2025.11.020 41380962

[B10] Helleday T. Petermann E. Lundin C. Hodgson B. Sharma R. A. (2008). DNA repair pathways as targets for cancer therapy. Nat. Rev. Cancer 8 (3), 193–204. 10.1038/nrc2342 18256616

[B11] Kerr S. Greenland S. Jeffrey K. Millington T. Bedston S. Ritchie L. (2023). Understanding and reporting odds ratios as rate-ratio estimates in case-control studies. J. Glob. Health 13, 04101. 10.7189/jogh.13.04101 37712381 PMC10502767

[B12] Konstantinopoulos P. A. Matulonis U. A. (2023). Clinical and translational advances in ovarian cancer therapy. Nat. Cancer 4 (9), 1239–1257. 10.1038/s43018-023-00617-9 37653142

[B13] Konstantinopoulos P. A. Waggoner S. Vidal G. A. Mita M. Moroney J. W. Holloway R. (2019). Single-arm phases 1 and 2 trial of niraparib in combination with pembrolizumab in patients with recurrent platinum-resistant ovarian carcinoma. JAMA Oncol. 5 (8), 1141–1149. 10.1001/jamaoncol.2019.1048 31194228 PMC6567832

[B14] Kurtz J. E. Pujade-Lauraine E. Oaknin A. Belin L. Leitner K. Cibula D. (2023). Atezolizumab combined with Bevacizumab and platinum-based therapy for platinum-sensitive ovarian cancer: Placebo-controlled randomized phase III ATALANTE/ENGOT-ov29 trial. J. Clin. Oncol. 41 (30), 4768–4778. 10.1200/jco.23.00529 37643382 PMC10602539

[B15] Lafargue C. J. Dal Molin G. Z. Sood A. K. Coleman R. L. (2019). Exploring and comparing adverse events between PARP inhibitors. Lancet Oncol. 20 (1), e15–e28. 10.1016/s1470-2045(18)30786-1 30614472 PMC7292736

[B16] Lheureux S. Braunstein M. Oza A. M. (2019a). Epithelial ovarian cancer: evolution of management in the era of precision medicine. CA Cancer J. Clin. 69 (4), 280–304. 10.3322/caac.21559 31099893

[B17] Lheureux S. Gourley C. Vergote I. Oza A. M. (2019b). Epithelial ovarian cancer. Lancet 393 (10177), 1240–1253. 10.1016/s0140-6736(18)32552-2 30910306

[B18] Liu J. F. Herold C. Gray K. P. Penson R. T. Horowitz N. Konstantinopoulos P. A. (2019). Assessment of combined nivolumab and bevacizumab in relapsed ovarian cancer: a phase 2 clinical trial. JAMA Oncol. 5 (12), 1731–1738. 10.1001/jamaoncol.2019.3343 31600397 PMC6802049

[B19] Marth C. Reimer D. Zeimet A. G. (2017). Front-line therapy of advanced epithelial ovarian cancer: standard treatment. Ann. Oncol. 28 (Suppl. l_8), viii36–viii39. 10.1093/annonc/mdx450 29232473

[B20] Matulonis U. A. Sood A. K. Fallowfield L. Howitt B. E. Sehouli J. Karlan B. Y. (2016). Ovarian cancer. Nat. Rev. Dis. Prim. 2, 16061. 10.1038/nrdp.2016.61 27558151 PMC7290868

[B21] Monk B. J. Lorusso D. Fujiwara K. Sehouli J. (2025). Optimal bevacizumab treatment strategy in advanced ovarian cancer: a review. Cancer Treat. Rev. 137, 102945. 10.1016/j.ctrv.2025.102945 40349571

[B22] Moore K. Colombo N. Scambia G. Kim B. G. Oaknin A. Friedlander M. (2018). Maintenance olaparib in patients with newly diagnosed advanced ovarian cancer. N. Engl. J. Med. 379 (26), 2495–2505. 10.1056/NEJMoa1810858 30345884

[B23] Ngoi N. Y. L. Leo E. O'Connor M. J. Yap T. A. (2021). Development of next-generation Poly(ADP-Ribose) polymerase 1-Selective inhibitors. Cancer J. 27 (6), 521–528. 10.1097/ppo.0000000000000556 34904816

[B24] Noguchi Y. Aoyama K. Kubo S. Tachi T. Teramachi H. (2020). Improved detection criteria for detecting drug-drug interaction signals using the proportional reporting ratio. Pharm. (Basel) 14 (1), 4. 10.3390/ph14010004 33374503 PMC7822185

[B25] O'Malley D. M. Krivak T. C. Kabil N. Munley J. Moore K. N. (2023). PARP inhibitors in ovarian cancer: a review. Target Oncol. 18 (4), 471–503. 10.1007/s11523-023-00970-w 37268756 PMC10344972

[B26] Peres L. C. Cushing-Haugen K. L. KöBEL M. Harris H. R. Berchuck A. Rossing M. A. (2019). Invasive epithelial ovarian cancer survival by histotype and disease stage. J. Natl. Cancer Inst. 111 (1), 60–68. 10.1093/jnci/djy071 29718305 PMC6335112

[B27] Perren T. J. Swart A. M. Pfisterer J. Ledermann J. A. Pujade-Lauraine E. Kristensen G. (2011). A phase 3 trial of bevacizumab in ovarian cancer. N. Engl. J. Med. 365 (26), 2484–2496. 10.1056/NEJMoa1103799 22204725

[B28] Pignata S. Lorusso D. Joly F. Gallo C. Colombo N. Sessa C. (2021). Carboplatin-based doublet plus bevacizumab beyond progression *versus* carboplatin-based doublet alone in patients with platinum-sensitive ovarian cancer: a randomised, phase 3 trial. Lancet Oncol. 22 (2), 267–276. 10.1016/s1470-2045(20)30637-9 33539744

[B29] Rawal K. B. Mateti U. V. Shetty V. Shastry C. S. Unnikrishnan M. K. Shetty S. (2023). Development of evidence-based indicators for the detection of drug-related problems among ovarian cancer patients. Front. Pharmacol. 14, 1203648. 10.3389/fphar.2023.1203648 37456735 PMC10348894

[B30] Ray-Coquard I. Pautier P. Pignata S. PéROL D. GonzáLEZ-MartíN A. Berger R. (2019). Olaparib plus bevacizumab as first-line maintenance in ovarian cancer. N. Engl. J. Med. 381 (25), 2416–2428. 10.1056/NEJMoa1911361 31851799

[B31] Ray-Coquard I. Leary A. Pignata S. Cropet C. GonzáLEZ-MartíN A. Marth C. (2023). Olaparib plus bevacizumab first-line maintenance in ovarian cancer: final overall survival results from the PAOLA-1/ENGOT-ov25 trial. Ann. Oncol. 34 (8), 681–692. 10.1016/j.annonc.2023.05.005 37211045

[B32] Reid B. M. Permuth J. B. Sellers T. A. (2017). Epidemiology of ovarian cancer: a review. Cancer Biol. Med. 14 (1), 9–32. 10.20892/j.issn.2095-3941.2016.0084 28443200 PMC5365187

[B33] Tada K. Maruo K. Isogawa N. Yamaguchi Y. Gosho M. (2020). Borrowing external information to improve Bayesian confidence propagation neural network. Eur. J. Clin. Pharmacol. 76 (9), 1311–1319. 10.1007/s00228-020-02909-w 32488331

[B34] Torre L. A. Trabert B. Desantis C. E. Miller K. D. Samimi G. Runowicz C. D. (2018). Ovarian cancer statistics, 2018. CA Cancer J. Clin. 68 (4), 284–296. 10.3322/caac.21456 29809280 PMC6621554

[B35] Yang C. Song X. Sun H. Chen X. Liu C. Chen M. (2025a). Cardiovascular adverse events associated with PARP inhibitors for ovarian cancer: a real world study (RWS) with Bayesian disproportional analysis based on the FDA adverse event reporting system (FAERS). Expert Opin. Drug Saf. 24 (9), 1039–1046. 10.1080/14740338.2024.2390640 39132853

[B36] Yang C. Song X. Sun H. Chen X. Liu C. Yang Y. (2025b). Peripheral neuropathy in patients with ovarian cancer administrating poly ADP-ribose polymerase inhibitors: a real-world study based on Bayesian disproportionality analysis of the US food and drug administration adverse event reporting system. Clin. Ther. 47 (9), 673–680. 10.1016/j.clinthera.2025.04.016 40413120

[B37] Zhang H. Zhou Z. Wang J. Wang S. Ren J. Zhang M. (2025a). Adverse drug reaction assessment of pembrolizumab in cervical cancer treatment: a real-world pharmacovigilance study using the FAERS database. Front. Immunol. 16, 1582050. 10.3389/fimmu.2025.1582050 40264768 PMC12011867

[B38] Zhang M. Zhu J. Bao Y. Ao Q. Mao X. Qiu Z. (2025b). Bevacizumab in ovarian cancer therapy: current advances, clinical challenges, and emerging strategies. Front. Bioeng. Biotechnol. 13, 1589841. 10.3389/fbioe.2025.1589841 40474872 PMC12138263

