# Supplementary material for: Adverse events associated with targeted therapy and immunotherapy for ovarian cancer: a FAERS pharmacovigilance study
Source: Front Pharmacol. 2026 Jun 23;17:1867038. doi: 10.3389/fphar.2026.1867038 (PMC13337649; doi:10.3389/fphar.2026.1867038)
Supplement: Supplementary file 1 [file Supplementaryfile1.docx]

**Supplementary Table 1**. The identification information about ovarian cancer contained in the indication file.

| Identification information of ovarian cancer |
| --- |
| Ovarian cancer, Ovarian cancer stage I, Ovarian cancer stage II, Ovarian cancer stage III, Ovarian germ cell cancer stage III, Ovarian epithelial cancer stage III, Ovarian cancer recurrent, Ovarian epithelial cancer recurrent, Ovarian endometrioid carcinoma, Ovarian epithelial cancer, Borderline Serous Tumour Of Ovary, Borderline Ovarian Tumour, Mucinous Cystadenocarcinoma Ovary, Cystadenocarcinoma Ovary, Ovarian clear cell carcinoma, Ovarian cancer metastatic, Ovarian cancer stage IV, Ovarian germ cell cancer stage IV, Ovarian epithelial cancer stage IV, Ovarian neoplasm, Ovarian granulosa cell tumour, Ovarian germ cell tumour, Ovarian germ cell teratoma, Ovarian germ cell cancer, Ovarian germ cell embryonal carcinoma, Ovarian germ cell teratoma stage III, Ovarian germ cell tumour mixed, Ovarian dysgerminoma, Ovarian dysgerminoma stage I, Serous cystadenocarcinoma ovary, Homologous recombination deficiency positive advanced ovarian cancer, Ovarian germ cell endodermal sinus tumour, and HER2 positive ovarian cancer. |

**Supplementary Table 2**. The top 20 most frequently reported safety signals related to PARP inhibitors based on ROR values at PT level.

| PT | Number | ROR (95%CI) | PRR (χ^2^) | IC (IC025) |
| --- | --- | --- | --- | --- |
| Energy increased | 58 | 23.08 (3.2-166.64) | 23.07 (19.47) | 2.35 (2.04) |
| Vitamin D decreased | 55 | 21.89 (3.03-158.16) | 21.88 (18.29) | 2.37 (2.05) |
| Nocturia | 51 | 20.29 (2.8-146.86) | 20.29 (16.71) | 2.39 (2.06) |
| Intentional underdose | 562 | 18.7 (10.55-33.12) | 18.63 (195.26) | 2.23 (2.12) |
| Hunger | 44 | 17.51 (2.41-127.08) | 17.5 (13.96) | 2.45 (2.1) |
| Brain neoplasm | 43 | 17.11 (2.36-124.25) | 17.11 (13.56) | 2.46 (2.1) |
| Multiple allergies | 39 | 15.52 (2.13-112.95) | 15.51 (12) | 2.51 (2.13) |
| Product dose omission | 583 | 14.55 (8.85-23.91) | 14.49 (194.94) | 2.27 (2.17) |
| Product dose omission in error | 464 | 14.24 (8.21-24.71) | 14.2 (154.24) | 2.28 (2.17) |
| Accidental underdose | 101 | 13.4 (4.25-42.25) | 13.39 (32.18) | 2.37 (2.13) |
| Dry throat | 64 | 12.73 (3.12-52.03) | 12.73 (19.74) | 2.45 (2.15) |
| Eastern Cooperative Oncology Group performance status | 31 | 12.33 (1.68-90.35) | 12.33 (8.88) | 2.65 (2.22) |
| Blood count abnormal | 488 | 12.17 (7.4-20.02) | 12.13 (157.39) | 2.31 (2.2) |
| Initial insomnia | 90 | 11.94 (3.78-37.72) | 11.93 (27.87) | 2.41 (2.16) |
| Red blood cell count increased | 30 | 11.94 (1.63-87.53) | 11.93 (8.5) | 2.67 (2.24) |
| Nightmare | 28 | 11.14 (1.52-81.88) | 11.14 (7.72) | 2.73 (2.28) |
| Photosensitivity reaction | 414 | 11.01 (6.58-18.43) | 10.98 (130.34) | 2.34 (2.22) |
| Platelet count abnormal | 81 | 10.75 (3.39-34.02) | 10.74 (24.36) | 2.46 (2.19) |
| Product dose omission issue | 1860 | 10.25 (8.11-12.95) | 10.14 (582.56) | 2.34 (2.29) |
| Computerised tomogram abnormal | 149 | 9.89 (4.37-22.36) | 9.88 (44.86) | 2.42 (2.22) |

Note: PARPi, poly (ADP-ribose) polymerase inhibitors; ROR, reporting odds ratio; PRR, proportional reporting ratio; IC, information content; PT, the preferred term.

**Supplementary Table 3**. The top 20 most frequently reported safety signals associated with bevacizumab based on ROR values at PT level.

| PT | Number | ROR (95%CI) | PRR (χ^2^) | IC (IC025) |
| --- | --- | --- | --- | --- |
| Gastrointestinal perforation | 86 | 71.91 (42.73-121.02) | 71.47 (974.37) | 0.29 (0.03) |
| Proteinuria | 160 | 34.64 (25.99-46.16) | 34.25 (1499.52) | 0.3 (0.11) |
| Intestinal perforation | 64 | 15.66 (10.97-22.35) | 15.59 (408.28) | 0.35 (0.06) |
| Embolism | 42 | 12.66 (8.35-19.2) | 12.63 (230.99) | 0.38 (0.02) |
| Hypokalaemia | 58 | 7.09 (5.17-9.72) | 7.06 (197.14) | 0.45 (0.14) |
| Impaired healing | 23 | 6.92 (4.2-11.4) | 6.91 (73.95) | 0.49 (0) |
| Lymphocyte count decreased | 35 | 6.44 (4.31-9.6) | 6.42 (106.25) | 0.49 (0.08) |
| Neurotoxicity | 25 | 6 (3.75-9.57) | 5.99 (69.26) | 0.52 (0.05) |
| Myelosuppression | 155 | 5.91 (4.9-7.13) | 5.86 (438.63) | 0.47 (0.28) |
| Deep vein thrombosis | 38 | 5.72 (3.93-8.35) | 5.71 (101.67) | 0.51 (0.12) |
| Hypertensive crisis | 20 | 5.66 (3.37-9.51) | 5.65 (51.21) | 0.55 (0.02) |
| Hypomagnesaemia | 29 | 5.62 (3.66-8.65) | 5.61 (75.22) | 0.52 (0.09) |
| General physical health deterioration | 61 | 5.62 (4.17-7.56) | 5.6 (161.6) | 0.5 (0.19) |
| Chronic kidney disease | 22 | 5.56 (3.39-9.1) | 5.55 (55.5) | 0.54 (0.04) |
| Epistaxis | 99 | 5.29 (4.2-6.67) | 5.26 (245.91) | 0.51 (0.26) |
| Polyneuropathy | 17 | 5.01 (2.88-8.71) | 5 (37.13) | 0.59 (0.03) |
| Hyponatraemia | 56 | 4.99 (3.68-6.77) | 4.98 (128.57) | 0.53 (0.21) |
| Hyperkalaemia | 16 | 4.92 (2.78-8.69) | 4.91 (33.98) | 0.6 (0.02) |
| Neoplasm malignant | 38 | 4.85 (3.35-7.01) | 4.84 (83.18) | 0.55 (0.17) |
| Thrombotic microangiopathy | 18 | 4.8 (2.81-8.2) | 4.8 (37.43) | 0.6 (0.05) |

Note: ROR, reporting odds ratio; PRR, proportional reporting ratio; IC, information content; PT, the preferred term.

**Supplementary Table 4**. Test for multicollinearity among variables in the multivariate logistic regression model.

| Variables | GVIF | Df | GVIF^(1/(2*Df)) |
| --- | --- | --- | --- |
| Age | 1.28 | 3 | 1.04 |
| Weight | 1.31 | 3 | 1.05 |
| Occupation_type | 1.97 | 6 | 1.06 |
| Reporter_country | 2.41 | 5 | 1.09 |
| Stage | 1.04 | 3 | 1.01 |
| Histological_subtype | 1.22 | 3 | 1.03 |
| Recurrent | 1.09 | 1 | 1.05 |
| Drug_type_all_2 | 1.52 | 6 | 1.04 |

Note: GVIF, generalized variance inflation factor; Df, degrees of freedom.

If GVIF^(1/(2*Df)) < 5, it indicates the absence of multicollinearity.


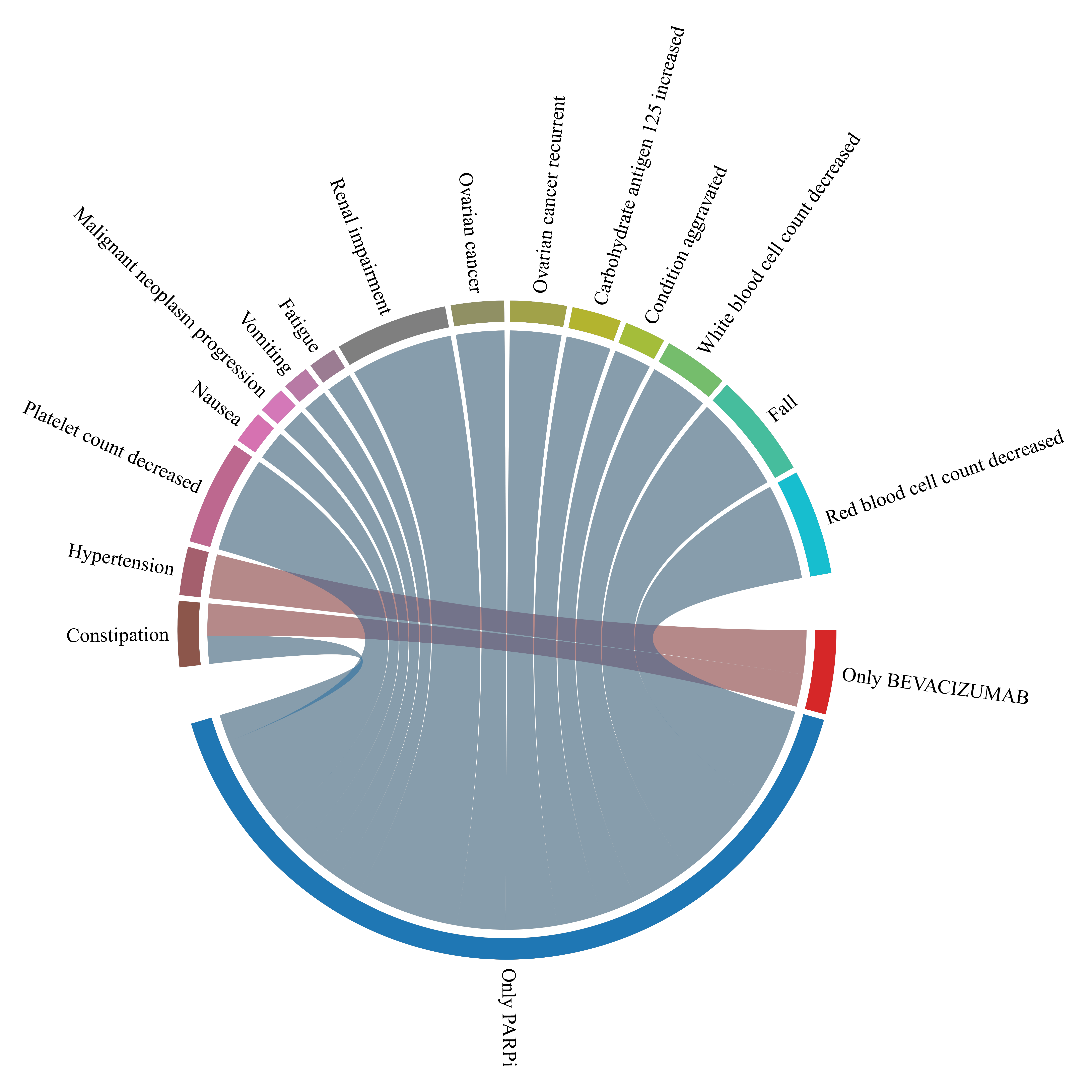


**Supplementary Figure 1**. The correspondence between drug combinations and adverse events in stage III ovarian cancer patients. The lower half of the figure represents individual and combined treatments, while the upper half represents associated PTs. The connecting lines between the upper and lower halves indicate significant signals generated by treatment-associated PTs. Different treatment regimens and associated PTs are distinguished by color, and each associated PT has connecting lines extending from at least two treatment regimens. PARPi, poly (ADP-ribose) polymerase inhibitors; PT, the preferred term.


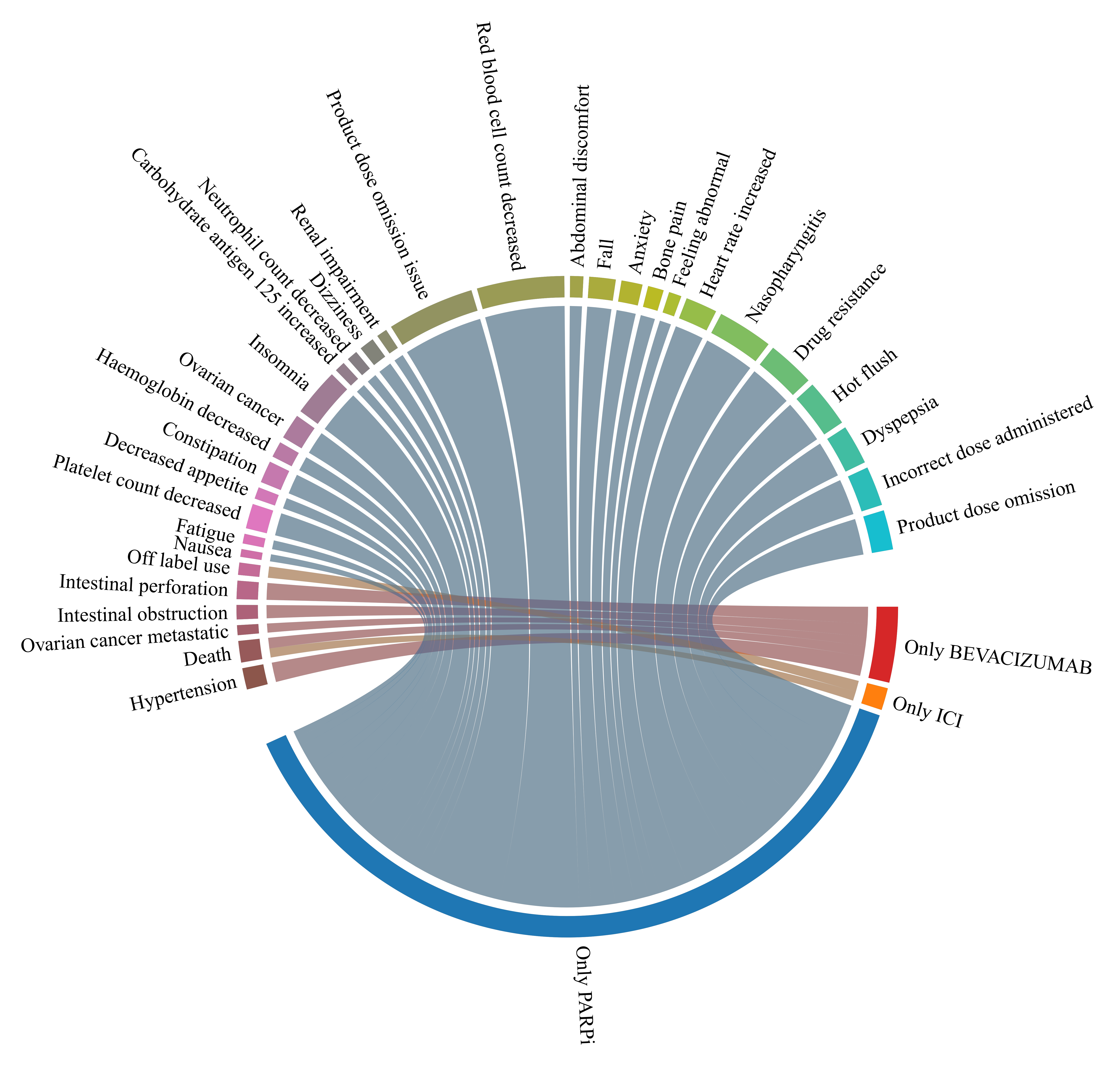


**Supplementary Figure 2**. The correspondence between drug combinations and adverse events in stage IV ovarian cancer patients. The lower half of the figure represents individual and combined treatments, while the upper half represents associated PTs. The connecting lines between the upper and lower halves indicate significant signals generated by treatment-associated PTs. Different treatment regimens and associated PTs are distinguished by color, and each associated PT has connecting lines extending from at least two treatment regimens. ICI, immune checkpoint inhibitors; PARPi, poly (ADP-ribose) polymerase inhibitors; PT, the preferred term.


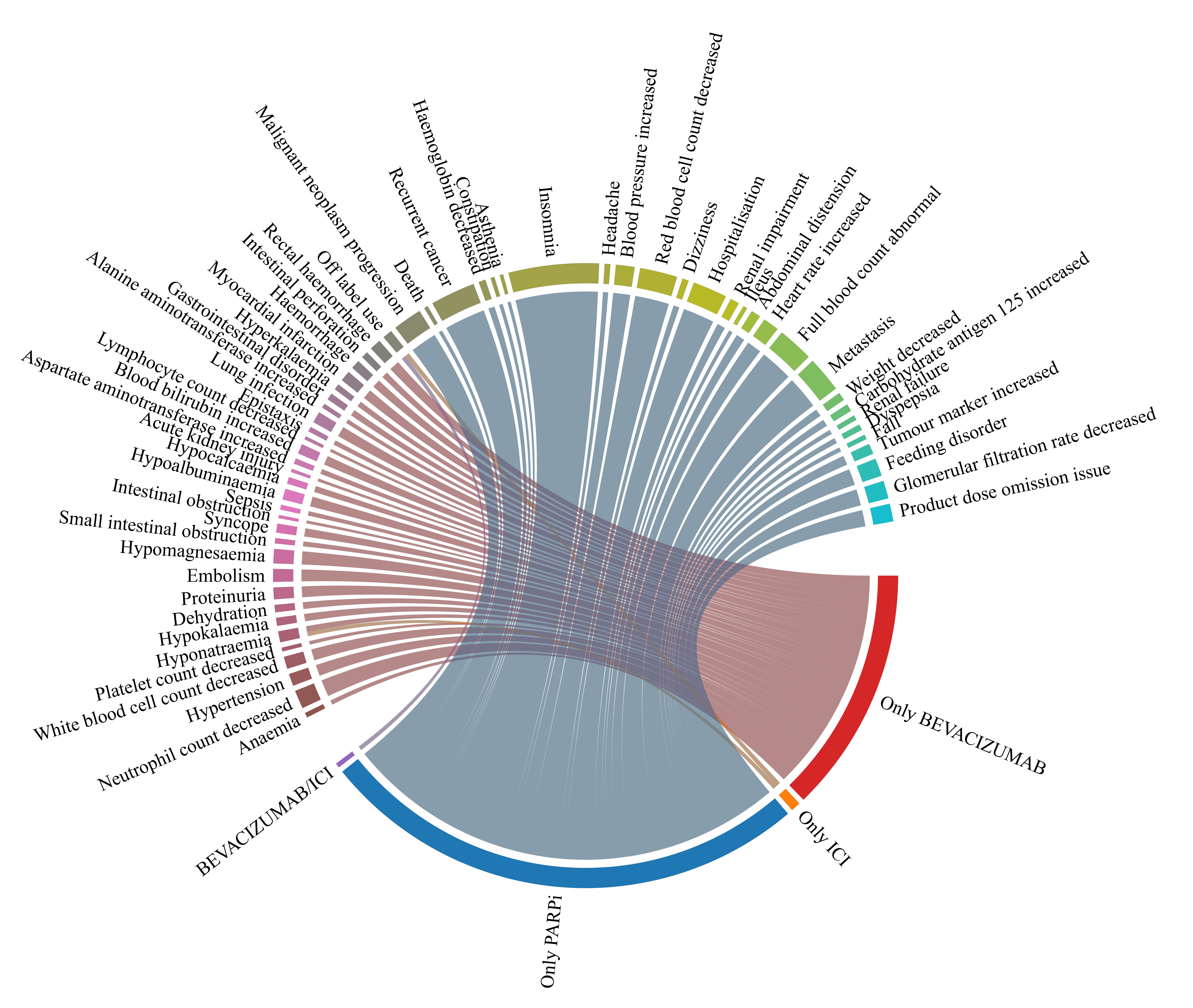


**Supplementary Figure 3**. The correspondence between drug combinations and adverse events in ovarian epithelial cancer patients. The lower half of the figure represents individual and combined treatments, while the upper half represents associated PTs. The connecting lines between the upper and lower halves indicate significant signals generated by treatment-associated PTs. Different treatment regimens and associated PTs are distinguished by color, and each associated PT has connecting lines extending from at least two treatment regimens. ICI, immune checkpoint inhibitors; PARPi, poly (ADP-ribose) polymerase inhibitors; PT, the preferred term.


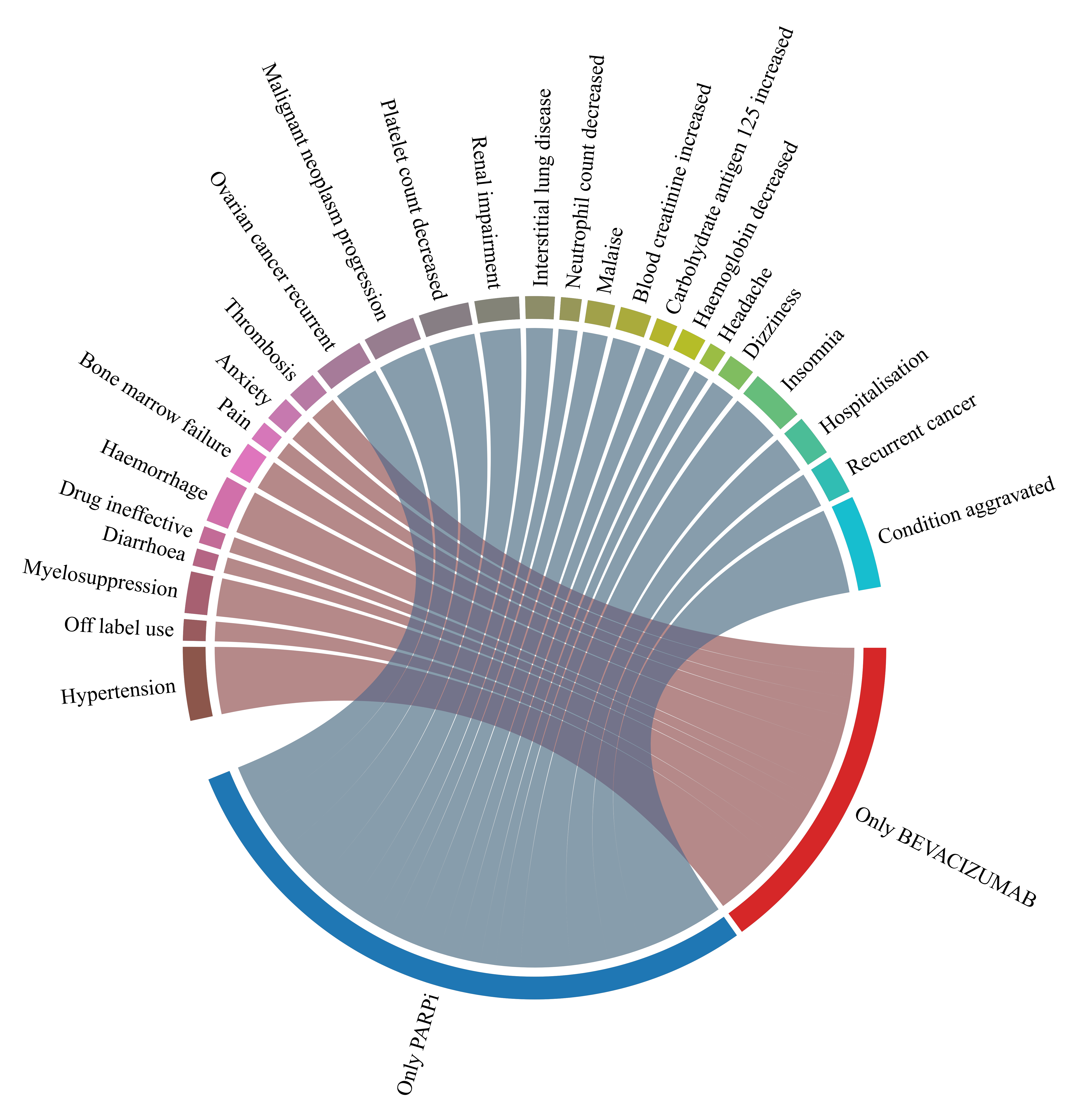


**Supplementary Figure 4**. The correspondence between drug combinations and adverse events in patients with recurrent ovarian cancer. The lower half of the figure represents individual and combined treatments, while the upper half represents associated PTs. The connecting lines between the upper and lower halves indicate significant signals generated by treatment-associated PTs. Different treatment regimens and associated PTs are distinguished by color, and each associated PT has connecting lines extending from at least two treatment regimens. PARPi, poly (ADP-ribose) polymerase inhibitors; PT, the preferred term.


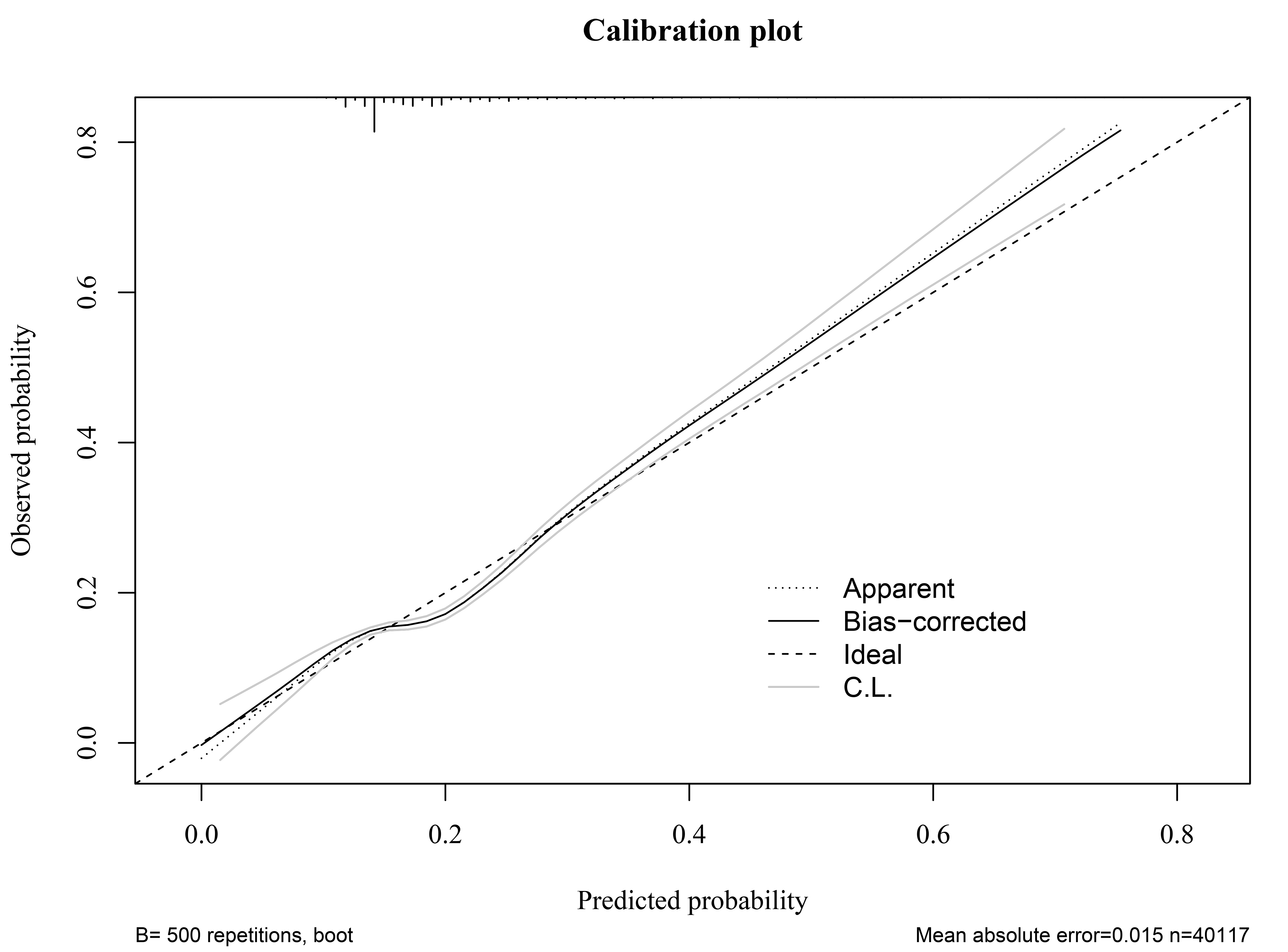


(A)


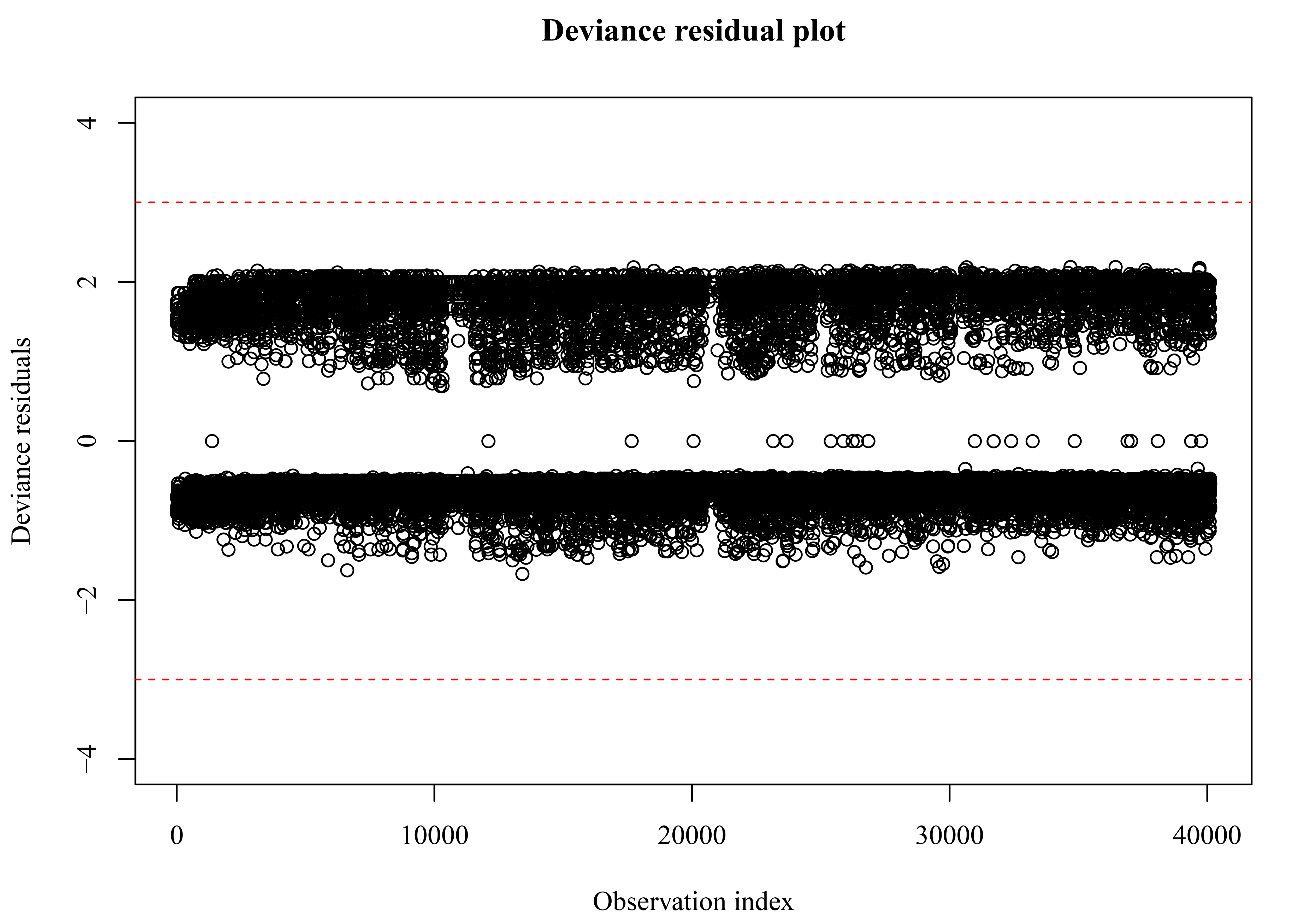


(B)

**Supplementary Figure 5**. Calibration curves and deviance residual plot of the multivariate logistic regression model. (A) Calibration curves; (B) Deviance residual plot.
